# Study on Fermentation Preparation, Stability, and Angiotensin-Converting Enzyme Inhibitory Activity of Tomato Pomace Peptide

**DOI:** 10.3390/foods14020145

**Published:** 2025-01-07

**Authors:** Ying Mu, Ruxianguli Maimaitiyiming, Jingyang Hong, Yu Wang, Yao Zhao, Ruoqing Liu, Liang Wang, Keping Chen, Aihemaitijiang Aihaiti

**Affiliations:** 1 College of Life Science and Technology, Xinjiang University, Urumqi 830000, China; muying2208@126.com (Y.M.); rm725276492@126.com (R.M.); hongjingyang2017@126.com (J.H.); wangyu170222@163.com (Y.W.); agneslll@163.com (Y.Z.); liuruoqing6688@163.com (R.L.); wl1390593786@163.com (L.W.); 2 Xinjiang Huize Food Limited Liability Company, Urumqi 830000, China; clnb188@126.com

**Keywords:** tomato pomace, two-stage fermentation, peptide, stability, structure characterization, ACE inhibitory activity

## Abstract

The substantial quantity of discarded tomato pomace (TP) results in the waste of valuable resources. This study utilizes these tomato by-products by mixing them with water in a specific proportion and fermenting the mixture in two stages: first with yeast, and then with lactic acid bacteria. The most suitable microbial strains for TP fermentation were identified by evaluating parameters such as peptide content, degree of hydrolysis, and gel electrophoresis analysis. Subsequently, tomato pomace peptides (TPPs) were separated into peptides of different molecular weights using ultrafiltration. The IC_50_ values, ACE inhibitory activities, and in vitro stability of these peptides were compared, and their secondary structures and microstructures were characterized. The results indicated that the soluble protein concentration increased from 26.25 mg/g to 39.03 mg/g after 32 h of fermentation with strain *RV171*. After an additional 32 h of fermentation with *Bifidobacterium thermophilum*, the peptide content reached 49.18 ± 0.43%. SDS-PAGE gel electrophoresis showed that the TPP molecular weights were predominantly below 10 kDa. The IC_50_ results demonstrated that fractions with smaller molecular weights exhibited greater ACE inhibitory activities. Structural analysis confirmed that the TP hydrolysate was indeed a peptide.

## 1. Introduction

Plant-derived peptides are small-molecule peptides formed by the hydrolysis of plant proteins under specific conditions [1]. These peptides exhibit unique biological functions that traditional proteins and amino acids do not possess, including antihypertensive [2], antioxidant [3], antidiabetic [4], and anticancer [5] effects. Studies have reported that peptides extracted from plant materials such as soybean, rapeseed, corn, and rice bran possess significant ACE inhibitory activity [6]. Angiotensin-converting enzyme (ACE), a dipeptide carboxypeptidase, is a key target in the pathogenesis of hypertension and has significant implications for human health [7]. ACE functioned to convert angiotensin I into the potent vasoconstrictor angiotensin II in the renin–angiotensin system (RAS) by cleaving the His–Leu dipeptide and inactivating the potent vasodilator bradykinin in the kallikrein–kinin system (KKS). Thus, the inhibition of ACE activity decreased the levels of angiotensin II and increased the levels of bradykinin, which was an effective way to reduce blood pressure [8]. In recent years, bioactive peptides exhibiting ACE inhibitory activity have been identified as being released from various plant-derived proteins; these peptides exhibited structural features similar to those of drug-like ACE inhibitors [9]. Their favorable antihypertensive effects, coupled with their non-toxicity and lack of side effects, position these natural ACE inhibitory peptides as promising alternatives to traditional ACE inhibitors. However, selecting suitable raw materials for the production of ACE inhibitory peptides remains a challenging task. Research has demonstrated that certain plant seeds and agricultural by-products rich in high-quality proteins, including green tea pomace [10], olive pomace [11], walnut meal [12], and coffee residue [13], possess significant potential for generating ACE inhibitory peptides.

According to Eurostat data from 2016, more than 10 million tonnes of the 17.9 million tonnes of tomatoes produced globally are used in the production of tomato paste, tomato juice, and canned tomatoes. A significant amount of TP is generated during the processing of tomato products, primarily consisting of peel and seeds (22%), along with a small portion of pulp, which accounts for approximately 5% to 10% of the total virgin tomato [12]. Several studies report that tomato seeds, containing approximately 20–40% protein by dry weight, are a rich source of nutrients and a high-quality protein resource [14]. Consequently, the underutilization of tomato pomace represents a significant waste of this valuable resource. To effectively utilize TP, studies have been conducted using heat-tolerant bacteria to produce lactic acid through the fermentation of both tomato pomace and apple pomace, thereby promoting the economic cycle [15]. However, this method did not fully utilize the protein resources, particularly the bioactive peptides, in tomato pomace. To enhance protein utilization, researchers have extracted bioactive peptides using water solubilization and acid precipitation [16]. However, this process often involves harmful reagents and is unsuitable for large-scale industrial production. Therefore, there is a need for the development of a safe and effective method in order to use proteins in TP. Some researchers have used *Bacillus subtilis* (YY-1) and *Saccharomyces cerevisiae* (YY-2) to ferment the canola meal by hydrolyzing its proteins to obtain active peptides [17]. Researchers found that fermenting milk with *Kluyveromyces marxianus* resulted in significantly greater peptide diversity than fermentation with *Lactobacillus* [18]. In another study, researchers obtained bioactive peptides with strong antioxidant and antimicrobial properties by fermenting tomato by-products using *Bacillus subtilis* [19]. However, their focus was primarily on studying the peptides’ antioxidant and antimicrobial properties. They did not provide detailed information on the peptide yields or conduct in-depth investigations into their stability and structure. It is well known that the processing steps and conditions of peptides can alter their biological activities. As a result, for bioactive peptides to be used as active ingredients, it is crucial that they can be stabilized across various environmental systems and processing methods [20].

A two-stage fermentation method was employed in this study to screen for the most suitable yeast and lactic acid bacteria strains and to optimize processing conditions for TPP fermentation. Subsequently, the IC_50_ values and in vitro stability of peptides with different molecular weights were further investigated. Finally, UV spectroscopy, Fourier transform infrared spectroscopy, and scanning electron microscopy were used to analyze the secondary structure and microstructure of the obtained ACE inhibitory peptides. This study developed a high-yield ACE inhibitory peptide with antihypertensive efficacy using TP. This approach not only effectively reduces resource waste but also facilitates the value-added utilization of waste, providing an important theoretical foundation for the development of new natural antihypertensive peptides and enhancing the utilization of plant-derived proteins.

## 2. Materials and Methods

### 2.1. Materials

TP (local sales market in Hutubi County, Xinjiang, China), enzyme labeling machines, and centrifuges were purchased from Molecular Devices (Silicon Valley, CA, USA) and Jiangsu Jinyi Instrument Technology Co., Ltd. (Changzhou, China), respectively. The microscope is from Motic (Xiamen, China). *Saccharomyces cerevisiae EC-1118*, *Saccharomyces cerevisiae RV171*, *Saccharomyces cerevisiae BV818*, *Torulaspora delbrueckii*, *Pichia guilliermondii*, and *Hansenula uvarum* were purchased in a lyophilized form (Beijing Baio Bowei Biotechnology Co., Ltd., Beijing, China). Twelve commercial strains, *Lactobacillus fermentum* CICC 25124, *Lactobacillus reuteri* CICC 6123, *Lactobacillus rhamnosus* CICC 6164, *Pediococcus pentosaceus* CICC 21865, *Lactobacillus delbrueckii* subsp. *Bulgaricus* CICC 6098, *Lactobacillus acidophilus* CICC 6086, *Lactobacillus plantarum* CICC 25125, *Pediococcus acidilactici* bio-097553, *Bifidobacterium thermophilum* bio-04950, *Lactobacillus paracasei* subsp. *paracasei* bio-53142, *Bacillus subtilis* CICC 24713, and *Bulgaricus* CICC 6098, were provided by the China Industrial Microbial Strains Preservation and Management Center (Beijing, China). Seventeen amino acid standards, OPA, FMOC, and angiotensin-converting enzyme (ACE) were purchased from Sigma (Livonia, MI, USA). N-[3-(2-Furyl)acryloyl]-Phe-Gly-Gly (FAPGG) was purchased from Shenggong (Shanghai, China). N-(2-hydroxyethyl)piperazine-N′-2 -ethanesulfonic acid (HEPES) was purchased from Genuine Leaf Biologicals (Shanghai, China). All solvents and chemicals, including sodium chloride, potassium chloride, manganese chloride, ferric chloride, and copper chloride, were of analytical reagent grade or higher.

### 2.2. Preparation of Tomato Pomace Peptide

The dried tomato pomace base was initially pulverized and sieved using grinding equipment. The resulting powder was suspended in water at a ratio of 1:15 (*w*/*v*) and mixed thoroughly. Following sterilization at 85 °C, the mixture was cooled and maintained at ambient temperature. Lipase (100,000 U/g) and cellulase (50 U/mg) were added to the tomato pomace solution at concentrations of 0.1% *w*/*w*, based on the weight of the tomato pomace. Subsequently, the enzymes were inactivated at 85 °C after a 6 h incubation in a water bath at 50 °C. After cooling the tomato pomace solution to room temperature, yeast was introduced into the solution at an initial concentration of 1 × 10^7^ CFU/mL, followed by dynamic fermentation at 28 °C for a designated period. Subsequent to sterilization, an additional 1 × 10^7^ CFU/mL bacteria were added, and static fermentation was conducted at 37 °C for a specified duration. The peptide concentration was measured every 8 h throughout the fermentation process. After fermentation, the fermentation broth was ultrafiltered. It was then centrifuged at 5000 rpm for 10 min at 4 °C to separate peptides of different molecular weights. The resulting peptides were freeze-dried at −80 °C and stored at −20 °C.

### 2.3. Peptide Content Detection

Following Cotton’s [21] methodology, 1 mL of sample solution was combined with 1 mL of 15% (*w*/*w*) aqueous trichloroacetic acid (TCA). After mixing and a 10 min rest, the solution was centrifuged at 4000 rpm for 10 min. One milliliter of supernatant was then extracted, combined with 4 mL of biuret reagent, vortexed for 1 min, and allowed to stand for 30 min. A blank was concurrently prepared by mixing 1 mL of water with 4 mL of biuret reagent. Absorbance was measured at 540 nm, and the peptide concentration (C, mg/mL) in the hydrolysate was determined using the standard regression equation.

### 2.4. Kjeldahl Nitrogen Determination

Following the determination of the optimal fermentation time for each microbiome, the fermentation residues were dried, and their nitrogen concentration was determined using the Kjeldahl method (GB 5009.5-2016) [22], employing a protein conversion factor of 6.25. Sample supernatants, collected at various fermentation time points, were centrifuged at 4000 rpm for 10 min at 4 °C. The nitrogen concentration in the resulting fermentation broth was then measured, and the protein content was calculated from this nitrogen content [23].

### 2.5. Sodium Dodecyl Sulfate–Polyacrylamide Gel Electrophoresis (SDS-PAGE)

The sample preparation was slightly modified from that of Wang et al. [24]. Before loading, the sample was denatured by mixing it with loading buffer in an EP tube and heating it at 95 °C for 5 min. The sample was then either immediately placed on ice or stored at −20 °C for later analysis. Approximately 20 µL of each sample was loaded into each well for electrophoresis. Electrophoresis was performed using the warm flow method at a constant current of 30 mA and a voltage of 120 V for 2 to 3 h. The electrophoresis apparatus was maintained in an ice bath throughout the process.

### 2.6. Molecular Weight Distribution

The main operating parameters of the instrument are as follows [22]:

(1) Column: TSKgel 2000 SWXL 300 mm × 7.8 mm; mobile phase: acetonitrile/water/trifluoroacetic acid, 45/55/0.1 (*V*/*V*); detection: UV 220 nm; flow rate: 0.5 mL/min; column temperature: 30 °C.
(2) Sample preparation: A total of 100 mg of the sample was transferred to a 10 mL volumetric flask, diluted accordingly with a mobile phase, and filtered through a 0.45 μm microporous membrane.
(3) The sample solutions were analyzed using the chromatographic conditions described above. The data were processed with GPC software to determine the molecular weight distribution and corresponding phase separation of the peptides, including their distribution ranges.

### 2.7. Amino Acid Analysis

A 2 mL liquid sample was taken and placed in a 10 mL glass tube [22]. Subsequently, 2 mL of hydrochloric acid (containing 1% phenol) was added, and the tube was purged with nitrogen for 1 min, sealed, and hydrolyzed at 110 °C for 22 h. After hydrolysis, the mixture was removed, cooled, and diluted with water to a final volume of 50 mL. Then, 1 mL of the solution was subjected to nitrogen blow-drying at 95 °C, followed by the accurate addition of 1 mL of 0.01 M HCl to dissolve the residue. The resulting solution was filtered through a membrane for chromatographic analysis under the following conditions: ZORBAX Eclipse AAA column (4.6 × 75 mm, 3.5 μm); detection wavelengths: UV 338 nm (0 to 19 min) and 266 nm (19.01 to 25 min); mobile phase A: 40 mM sodium dihydrogen phosphate (pH 7.8); mobile phase B: a mixture of acetonitrile, methanol, and water in a ratio of 45:45:10; flow rate: 1.0 mL/min. The amino acid content was analyzed using an Agilent ODS column (5 μL, 4.0 mm × 250 mm). Gradient elution was conducted according to the following protocol: 0 min 0% B; 23 min 57% B; 27 min 100% B; 40 min 0% B. The mobile phase was analyzed using a UV detector (VWD) at a flow rate of 1.0 mL/min, with wavelengths set at 338 nm for general detection and 266 nm for proline. The amino acid composition was measured using the external standard method.

### 2.8. Purification of Tomato Pomace Peptides

Ultrafiltration of the fermentation broth using centrifuge tubes with cut-off values of 1 and 3 kDa yielded peptides of varying molecular weights. These peptides were subsequently freeze-dried and stored at −80 °C for later use.

### 2.9. Assessment of ACE Inhibition Rate In Vitro

N-[3-(2-Furyl)acryloyl]-Phe-Gly-Gly was utilized as a simulated substrate for angiotensin I. The reactants were added to a 96-well microtiter plate in the order shown in Table 1, and the absorbance (A1) at 340 nm was measured using a microplate reader prior to the reaction. The reactants in the microplate continued to react at 37 °C for 30 min, after which the absorbance (A2) was measured again [25]. Parallel experiments were conducted three times, and the results were expressed as the mean ± standard deviation.

ACE inhibition rate (%) = (1 − ΔAa/ΔAb) × 100 (1)

ΔAb was the change in absorbance within 30 min when the buffer matrix was added.

ΔAa was the change in the absorbance of the inhibitor within 30 min.

The ACE inhibition rate for each concentration gradient was determined using the presented equation, and the IC_50_ (the concentration of hydrolysate or peptide that inhibits ACE activity by 50%) value for ACE inhibition by the TPP was subsequently calculated using GraphPad Prism 5.02 software.

The following procedures should be noted for each reagent: FAPGG (1.0 mmol/L): Dissolve 3.994 mg FAPGG in matrix buffer to a final volume of 10 mL. Store at 4 °C in the dark. Matrix Buffer: Dissolve 1.910 g HEPES and 1.755 g NaCl in 100 mL of double-distilled water. Adjust the pH to 8.3 with NaOH. Store at 4 °C. ACE Solution: Dissolve ACE in 0.1 mol/L boric acid–borax buffer (pH 8.3) containing 0.3 mol/L NaCl.

### 2.10. Inhibition Stability

The stability of bioactive peptides is crucial for their bioactivity. Therefore, this study examined how temperature, pH, metal ions, in vitro simulated gastrointestinal digestion, organic solvents, and ionic strength affect the stability of tomato pomace-derived peptides.

#### 2.10.1. Investigation of Thermal Stability

Peptide solutions (2 mg/mL) of varying molecular weights were incubated in a water bath at 60, 70, 80, 90, and 100 °C for 2 h [26]. The samples were then rapidly cooled to room temperature in an ice-water bath before determining peptide content and ACE inhibitory activity.

#### 2.10.2. Evaluation of pH Stability

Peptide samples were dissolved in pH 2, 4, 6, 8, 10, 11, and 12 Na_2_HPO_4_ -citrate buffer (0.1 mol/L) and prepared to a mass concentration of 2 mg/mL. pH was adjusted with HCl and NaOH (1 mol/L), respectively, and the peptide content and ACE inhibitory activity were determined by standing for 2 h at room temperature [27].

#### 2.10.3. Stability of Organic Solvents

Peptide solutions (2 mg/mL) of varying molecular weights were prepared. Each solution was mixed with methanol, ethanol, and glycerol at concentrations of 10%, 20%, 30%, 40%, and 50%, respectively. After 2 h of oscillation, peptide content and ACE inhibitory activity were determined [25].

#### 2.10.4. Stability of Metal Ions

Tomato pomace peptide solutions (2 mg/mL) of varying molecular weights were prepared. Metal salts (K⁺, Mn^2^⁺, Fe^2^⁺, and Cu^2^⁺) were added at concentrations of 100, 150, 200, 250 and 350 μg/mL, respectively. The peptide content and ACE inhibitory activity were finally determined by standing for 2 h at room temperature.

#### 2.10.5. Stability of Ionic Concentration

A 2 mg/mL peptide solution was prepared. NaCl solutions (0.2, 0.4, 0.6, 0.8, and 1 mol/L) were added to create a concentration gradient. After a 2 h incubation, the supernatant was collected and analyzed for peptide content and ACE inhibition rate.

#### 2.10.6. Simulated Digestion Experiment In Vitro

A simulated digestion experiment in vitro was conducted according to the method of Chen et al. [28] with slight modifications. The 1 mg/mL TPP sample solution was mixed with the oral digestive solution at a volume ratio of 1:1 and oscillated at 37 °C and 200 r/min for 2 min, and 2 mol/L HCl was immediately added to adjust the pH to 2.0 to stop the oral simulated digestion. Subsequently, 10 mL of gastric juice was introduced and incubated for 2 h at 37 °C with a stirring rate of 200 revolutions per minute. After that, 2 mL of 2 mol/L NaOH was swiftly introduced to modify the pH to 7.0, concluding the simulated stomach digestion. Subsequently, 10 mL of simulated intestinal solution was added to the digestive system, consisting of 20 g of trypsin (4000 U/g), 172.2 mg of bile salts, and 355.2 mg of calcium chloride dissolved in 400 mL of phosphate buffer (pH 6.8) at final concentrations of 100 U/mL, 1 mM, and 4 mM, respectively. The mixture was then neutralized with sodium bicarbonate (1 M). Then, the mixtures were neutralized to pH 7.0 with sodium bicarbonate (1 M) and incubated at 200 rpm at 37 °C for 2 h. The enzymes were then inactivated by boiling in a water bath for 10 min. After centrifugation at 5000 rpm for 10 min, the supernatant was obtained to evaluate the effects of TPPs on ACE inhibition rate and peptide retention rate during gastrointestinal digestion.

### 2.11. Peptide Characterization

#### 2.11.1. Structural Analysis

The structural properties of peptides with varying molecular weights were analyzed using Fourier transform infrared (FT-IR) and ultraviolet–visible (UV-VIS) spectroscopy [29]. In a desiccated environment, samples visible to the naked eye and an appropriate amount of dry potassium bromide powder were combined in a mortar and thoroughly ground multiple times. The mixture was then placed into a tablet press to form translucent sheets. A background spectrum was collected prior to the analysis, followed by the recording of infrared spectra for 32 samples at a scanning resolution of 4 cm^−1^ within the wavelength range of 4000 to 400 cm^−1^. The substance was dissolved in distilled water at a concentration of 1.0 mg/mL and analyzed using a UV-2450 ultraviolet–visible absorption spectrophotometer (Shanghai, Mepheda). The scanning range was set from 200 to 400 nm, with distilled water serving as the blank control.

#### 2.11.2. Morphology Analysis

A trace of the sample was applied directly onto the conductive adhesive, followed by gold sputtering using the Quorum SC7620 sputtering coater (Quorum, Laughton, East Sussex, England) for 45 s (specific sputtering time may vary based on sample/testing requirements) at a current of 10 mA. Subsequently, the sample morphology was captured using the ZEISS Sigma300/ZEISS GeminiSEM 300/TESCAN MIRA LMS scanning electron microscope (SEM) (Zeiss, German). The accelerating voltage was set to 3 kV for topography imaging and 15 kV for mapping, utilizing the SE2 secondary electron detector.

### 2.12. Statistical Analysis

All results were shown as mean ± standard deviation (S.D.). The difference was assessed using a one-way analysis of variance, succeeded by Duncan’s range test.

## 3. Results and Discussion

### 3.1. Preparation of Peptides from Tomato Pomace Using a Two-Stage Fermentation Method

#### 3.1.1. Screening of Strains During the Yeast Fermentation Phase

**Figure 1:**
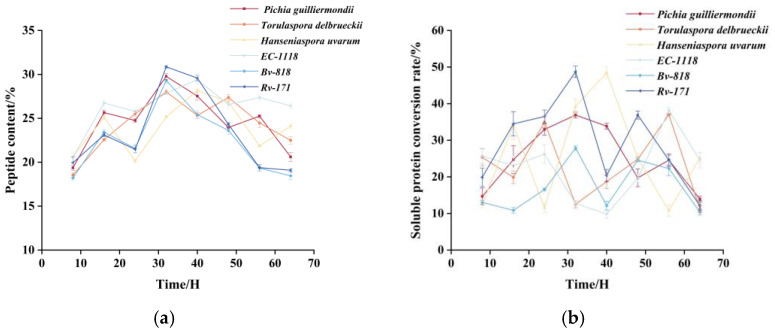

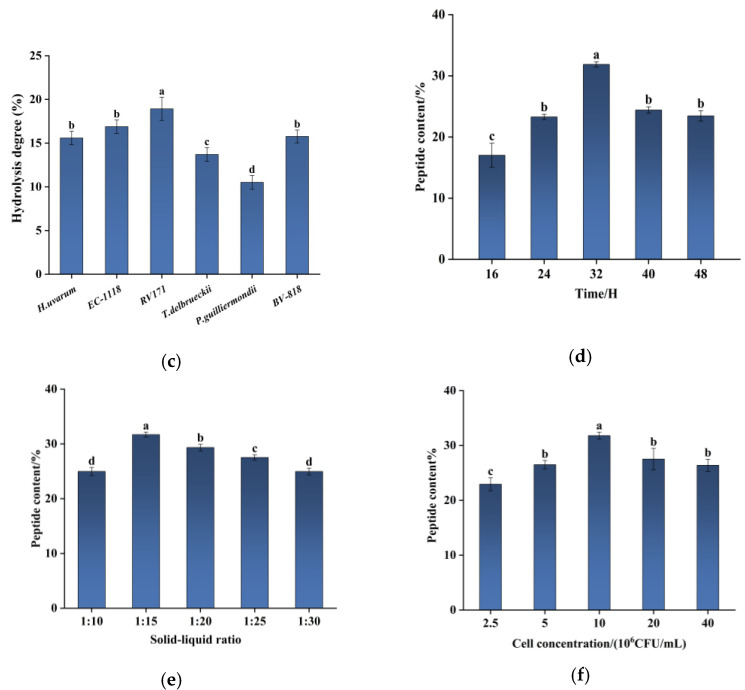
(**a**) Changes in peptide content (%) during the fermentation of TP using six different yeast strains. (**b**) Changes in the conversion of soluble protein (%) during the fermentation of tomato pomace by six different yeast strains. (**c**) Results of hydrolysis (%) of fermentation residues from different yeast strains are expressed as the mean ± standard deviation (SD) of three replicates. (**d**) Effect of fermentation time on peptide content (%). (**e**) Effect of the solid–liquid ratio on peptide content (%). (**f**) Effect of inoculum amount on peptide content (%). Lowercase letters indicate significance (*p* < 0.05).

Different fermentation strains yield distinct fermentation results (Figure 1a). Among the six yeast strains, *RV-171* exhibited the most effective fermentation, achieving the highest peptide content of 30.86 ± 0.14%. This result is comparable to that of Xinyu Heng et al. who utilized *Saccharomyces cerevisiae* in combination with multiple strains to ferment peptides, which increased the peptide content from 7.35% to 39.58 [30]. The *Hanseniaspora uvarum* strain showed the lowest fermentation performance, yielding a peptide content of 25.17 ± 0.14%. The peptide content of *Pichia guilliermondii*, *Torulaspora delbrueckii*, *EC-1118*, and *Bv-818* strains fluctuated consistently during fermentation, possibly due to the continual coalescence and dispersion of small-molecule peptides and amino acids [31].

A higher soluble protein content indicates that more proteins can be degraded into peptides during the fermentation process. At the onset of fermentation, the soluble protein content was 26.25 mg/g (Figure 1b). As fermentation time progressed, the soluble protein content in the supernatant continued to rise, and by 32 h, the soluble protein level of the *Rv171* strain had increased to 39.03 mg/g, representing a 48.68% increase compared to the initial value. This finding is consistent with the results of Huang et al. [32], likely due to fat degradation reducing protein–fat interactions and increasing protein solubility [33].

The degree of hydrolysis is a crucial parameter in protein hydrolysis, as it determines both the length of the peptide chain and the amino acid composition [34]. We analyzed the nitrogen content of the fermentation residues from six yeast strains at the highest peptide yield during fermentation and calculated their degree of hydrolysis. As illustrated in Figure 1c, the *Pichia guilliermondii* strain exhibited the lowest degree of hydrolysis at 10.51%, while the *RV-171* strain showed the highest degree at 18.93%. The remaining strains displayed a moderate degree of hydrolysis. These results suggest that the selected yeast strains did not sufficiently degrade the raw proteins, indicating that further lactic fermentation is necessary to enhance degradation.

The TPP produced by six yeast strains following fermentation was analyzed using sodium dodecyl sulfate–polyacrylamide gel electrophoresis (SDS-PAGE) (Figure 3a). Post-fermentation, the molecular weight distribution of TPPs was predominantly within the range of 10–130 kDa. Although the content of high-molecular-weight proteins exhibited a slight decrease, they were still present, suggesting that yeast fermentation facilitated peptide formation and enhanced the concentration of soluble proteins in the supernatant. Among the strains examined, *RV-171* demonstrated a significantly greater capacity for degrading tomato proteins compared to the other strains after fermentation. These findings indicate that strain *RV-171* possesses the highest fermentation potential.

#### 3.1.2. Single-Factor Optimization of Yeast Fermentation

The effects of the three factors on TPP content are illustrated in Figure 1d–f. The peptide content gradually increased with fermentation time, reaching a maximum of 31.47 ± 0.43% at 32 h before declining to 23.84 ± 0.84% (Figure 1d). This decline may be attributed to the deterioration of the culture environment, which is likely due to nutrient depletion, an imbalance in the carbon-to-nitrogen ratio, and changes in physicochemical factors [33]. Peptide content increased from 24.97 ± 0.74% to 31.7 ± 0.45% at solid–liquid ratios of 1:10 to 1:15, then decreased to 24.97 ± 0.59% at ratios above 1:15 (Figure 1e). This phenomenon likely results from lower solid–liquid ratios providing a higher proportion of nitrogen sources. This creates eutrophic conditions for the microorganisms, thus decreasing the rate of protein hydrolysis [35]. Conversely, excessively high solid–liquid ratios limit the availability of nutrients for microorganisms, inhibiting their growth and reproduction and consequently decreasing their peptide content [36]. Additionally, the peptide content reached 35.53% when the bacterial concentration was 1 × 10^7^ CFU/mL (Figure 1f). A comprehensive analysis indicated that the optimal fermentation conditions for the yeast strain were a solid–liquid ratio of 1:15, an initial bacterial density of 1 × 10^7^ CFU/mL, and a fermentation time of 32 h. Under these optimized conditions, the fermentation capacity of the *Saccharomyces cerevisiae RV-171* strain was maximized.

#### 3.1.3. Screening of Strains at the Fermentation Stage of Lactobacillus

**Figure 2:**
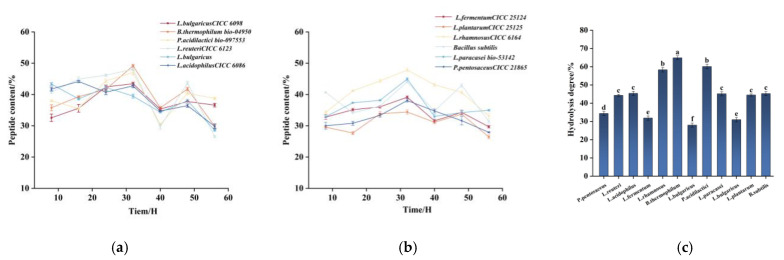
(**a**,**b**) The change in peptide content (%) during tomato pomace fermentation was assessed for each of the 12 bacterial taxa (TP). Data are presented as the mean ± standard deviation of three replicates. (**c**) The hydrolysis results (%) of different bacterial fermentation residues are presented as the mean ± standard deviation (SD) of three replicates. Lowercase letters denote statistical significance (*p* < 0.05).

*Lactobacillus* fermentation is an efficient and safe method for producing food-grade hydrolyzed peptides compared to yeast fermentation, and it plays a crucial role in regulating intestinal flora. Figure 2a,b illustrate that most strains reached their peak peptide content at 32 h, after which it either stabilized or declined. This phenomenon may be associated with the continuous condensation and dispersion of small-molecule peptides and amino acids during the fermentation process [37]. Among the 12 strains of lactic acid bacteria, *Bifidobacterium thermophilum* exhibited the highest peptide content at 49.18 ± 0.43%, a result that aligns with the findings of Ma et al. [38] who demonstrated the superior effect of *bifidobacterial* taxa in fermentation. *Lactobacillus plantarum* exhibited the lowest fermentation performance, with a peptide content of 26.45 ± 0.64%. At the peak of fermentation, the peptide content of *Pediococcus acidilactici* was 47 ± 0.84%, while *Bacillus subtilis* contents was 44.06 ± 0.35%, *Lactobacillus rhamnosus* produced 47.84 ± 0.54%, and *Lactobacillus paracasei* subsp. *Paracasei* had a peptide content of 44.96 ± 0.43%. The fermentation trends of *Lactobacillus delbrueckii* subsp. *Bulgaricus*, *Lactobacillus reuteri*, *Lactobacillus acidophilus*, *Lactobacillus fermentum*, and *Pediococcus pentosaceus* were similar. The strains used in this experiment are commonly employed in the fermentation of edible seeds and grains, producing fermentation products that are rich in bioactive small peptides with potential nutritional and health benefits.

In subsequent lactic fermentation studies, we assessed the degree of hydrolysis of the fermentation residues from different strains during the period of peak peptide content (Figure 2c). The results indicated that the hydrolysis degree of most strains exceeded 30%, with *Bifidobacterium thermophilum* exhibiting the highest hydrolysis degree of 64.90%. This suggests that tomato proteins were effectively broken down into peptides and free amino acids. By analyzing the data on peptide content and degree of hydrolysis, we found that *Bifidobacterium thermophilum*, *Pediococcus acidilactici*, *Lactobacillus paracasei* subsp. *paracasei*, *Lactobacillus reuteri*, *Bacillus subtilis*, and *Lactobacillus rhamnosus* exhibited the most favorable fermentation results after a fermentation period of 32 h.

**Figure 3:**
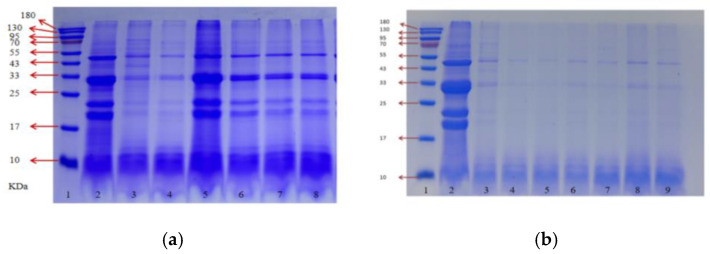
SDS-PAGE pattern of TP after fermentation: (**a**) 1—marker; 2—TP; 3—*Saccharomyces cerevisiae* BV818; 4—Saccharomyces cerevisiae RV171; 5—*Hanseniaspora uvarum*; 6—*Pichia guilliermondii*; 7—*Torulaspora delbrueckii*; 8—*Saccharomyces cerevisiae* EC 1118. (**b**) SDS-PAGE after lactic acid fermentation: 1—marker; 2—TP; 3—*Saccharomyces cerevisiae* RV171; 4—*Pediococcus acidilactici*; 5—lactobacillus rhamnosus; 6—*Lactobacillus paracasei* subsp. *paracasei*; 7—*Bacillus subtilis*; 8—*Lactobacillus reuteri*; 9—thermophilic bifidobacteria.

Figure 3b presents the SDS-PAGE results of fermented TPPs produced by different lactic acid bacteria. Compared to yeast fermentation, *Lactobacillus* fermentation more efficiently degraded large molecular tomato proteins into small molecular peptides, with the resulting fermented peptides primarily distributed below 10 kDa, which aligns with the findings of Bayat et al. [39]. Four dominant strains—*Bifidobacterium thermophilum*, *Pediococcus acidilactici*, *Lactobacillus rhamnosus*, and *Lactobacillus paracasei subsp. paracasei*—were selected for subsequent studies based on their peptide yield, degree of hydrolysis, and SDS-PAGE data analysis.

#### 3.1.4. Distribution of Molecular Weight

**Figure 4:**
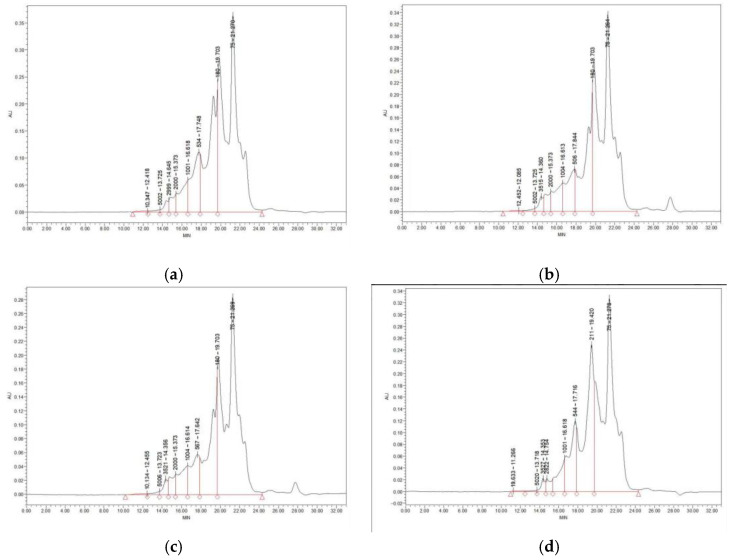
The molecular weight distribution of the fermented peptides derived from the four strains. (**a**) Molecular weight distribution of peptides fermented by *Bifidobacterium thermophilus*. (**b**) Molecular weight distribution of peptides derived from *Lactobacillus rhamnosus* fermentation. (**c**) Molecular weight distribution of peptides resulting from *Pediococcus acidilactici* fermentation. (**d**) Molecular weight distribution of peptides obtained from *Bacillus subtilis* fermentation.

The determination of molecular weight is crucial for studying the functional bioactivity of peptides [40]. As depicted in Figure 4 and Table 2, molecular weight determinations were performed on the four bacterial strains selected for lactic acid fermentation. The results indicated that the molecular weight distributions of the proteins produced by the four bacterial strains were highly comparable, predominantly concentrated below 1 kDa and within the range of 1–3 kDa. Notably, the proportion of peptides smaller than 3 kDa in *Bifidobacterium thermophilum* reached 98.35%, significantly exceeding that of the other three strains, thereby suggesting a superior fermentation capability compared to the other strains. In contrast, the percentages of peptides with molecular weights exceeding 3 kDa were relatively high in *Lactobacillus rhamnosus* and *Pediococcus acidilactici*, at 2.39% and 2.59%, respectively (Table 2).

Additionally, the molecular weight distribution of *Bacillus subtilis* exhibited similarities to that of *Bifidobacterium thermophilum*, with a predominance of peptides smaller than 3 kDa. This finding aligns with previous studies on the molecular weight distribution of wheat peptides following hydrolysis, indicating that lactic acid bacteria facilitate the degradation of large proteins into smaller peptides through the secretion of extracellular proteases during their growth, thereby enhancing their hydrophilicity [41]. Overall, the majority of fermented peptides generated by the four bacterial strains exhibited molecular weights below 1 kDa, indicating that the fermentation process effectively hydrolyzed proteins into peptides and amino acids. Although variations exist in the molecular weight distributions among the four bacterial strains, these differences may stem from the distinct types and quantities of proteases secreted by each strain during fermentation. This, in turn, influences the length of the peptide chains in the hydrolysate and the composition of their terminal amino acids [42].

#### 3.1.5. Amino Acid Content

A diverse array of proteases generated during microbial fermentation can effectively hydrolyze proteins and amino acids, thereby enhancing the amino acid content within the peptides [43]. Reports indicate that hydrophobic amino acid residues function as competitive inhibitors of ACE because they preferentially bind to the ACE catalytic site [44]. Amino acid analysis revealed that the concentration of hydrophobic amino acids in the supernatant after fermentation differed among various bacterial strains (Table 3), including *Bifidobacterium thermophilum* (3.5306 mg/mL), *Lactobacillus rhamnosus* (3.0649 mg/mL), *Bacillus subtilis* (2.9395 mg/mL), and *Pediococcus acidilactici* (2.5536 mg/mL).

The results indicate that glutamic acid (Glu) was particularly abundant in TPPs, with Glu concentrations in the fermentation broths of *Bifidobacterium thermophilum*, *Lactobacillus rhamnosus*, *Bacillus subtilis*, and *Pediococcus acidilactici* measured at 2.3965 mg/mL, 2.0211 mg/mL, 2.3134 mg/mL, and 2.2077 mg/mL, respectively. This finding aligns with the glutamic acid content reported by Han et al. in their study employing *Bacillus subtilis* for the fermentation of tomato waste [45]. Most potent antihypertensive peptides contain positively charged *C*-terminal amino acids, such as lysine and arginine. Arginine, notably, is also an important energy source for bacteria [46]. Following the fermentation treatment, the arginine content produced by the four bacterial strains was as follows: *Bifidobacterium thermophilum* (0.7414 mg/mL) > *Bacillus subtilis* (0.6355 mg/mL) > *Pediococcus acidilactici* (0.6251 mg/mL) > *Lactobacillus rhamnosus* (0.4917 mg/mL). By combining the results of the amino acid and molecular weight data, we found that *Bifidobacterium thermophilum* exhibited superior fermentation among the four strains. The amino acid composition of fermented products varies depending on the microbial community involved. This variation arises, in part, from the differential expression of enzymes by different microbial taxa during fermentation. Consequently, the resulting products exhibit diverse amino acid profiles and concentrations, leading to distinct biological effects [47].

### 3.2. Variations in the IC_50_ Values of TPPs Before and After Ultrafiltration

**Figure 5:**
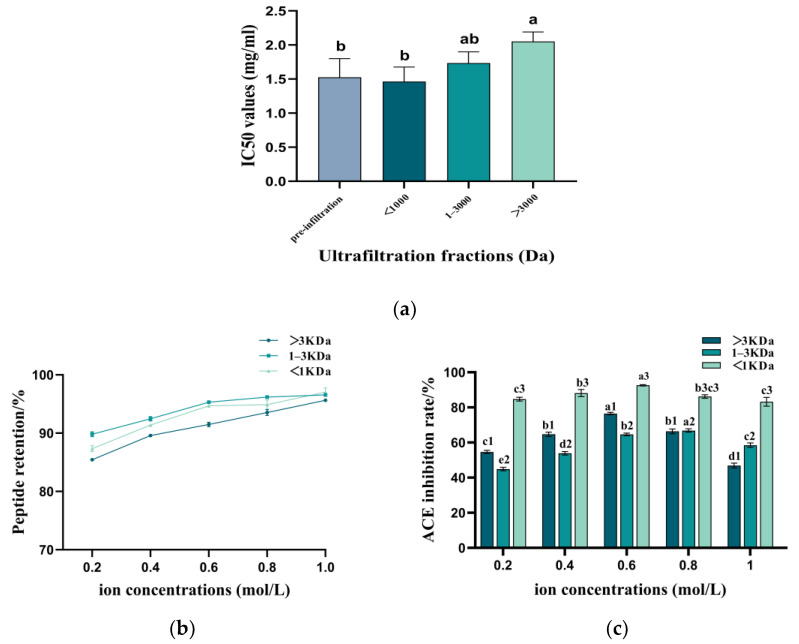

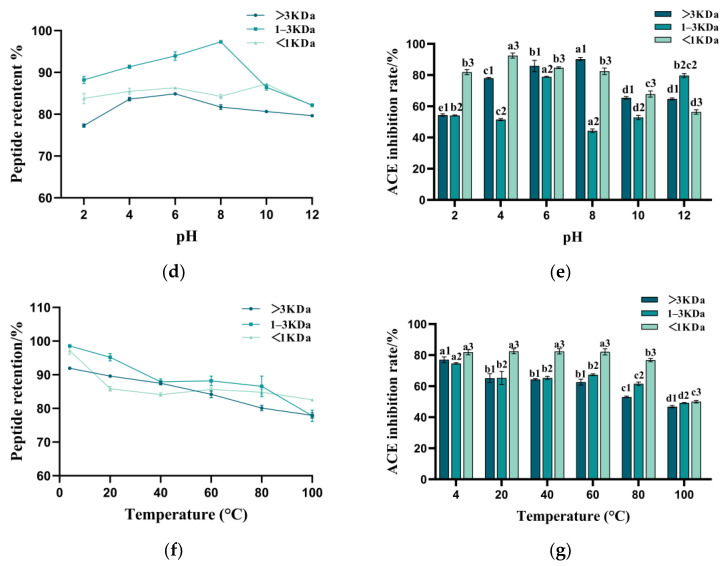
(**a**) Variations in ACE inhibitory IC_50_ values of TPPs before and after ultrafiltration. (**b**) Peptide retention in samples treated with different ion concentrations. (**c**) ACE inhibitory activity of samples treated with different ion concentrations. (**d**) Effect of different pH-treated samples on peptide content. (**e**) Effect of samples treated with different pH on ACE inhibition rates. (**f**) Effect of samples treated with different temperatures on peptide content. (**g**) Effect of samples treated with different temperatures on ACE inhibition rate. Different letters indicate statistically significant differences among treatments for each sample (*p* < 0.05).

Ultrafiltration significantly enhanced the ACE inhibitory activity of TPPs, as illustrated in Figure 5a. The IC_50_ value for the ACE inhibitory activity of the ultrafiltration fraction with a molecular weight of less than 1 kDa decreased to 1.46 mg/mL, representing a purification factor of 1.4 ± 0.21 compared to the pre-ultrafiltration TPP IC_50_ value of 1.5 ± 0.27 mg/mL. The IC_50_ values for the ACE inhibitory activity of fractions with molecular weights greater than 3 kDa and between 1 kDa and 3 kDa were 2.05 ± 0.14 mg/mL and 1.73 ± 0.16 mg/mL, respectively. Xu et al. [48] fractionated soybean protein hydrolysate into four components using ultrafiltration. The <3 kDa fraction exhibited significantly greater ACE inhibitory activity than the 3–5 kDa, 5–10 kDa, and >10 kDa fractions. Consistent with our findings, smaller-molecular-weight fractions showed superior ACE inhibition, likely due to improved bioavailability. This suggests that molecular weight is a key factor influencing ACE inhibitory activity.

### 3.3. Effect of Ionic Strength on the Stability of Peptides with Varying Molecular Weights

The concentration of NaCl significantly influenced the various components of TPPs (Figure 5b,c). As NaCl concentration increased, the retention of peptides of different molecular weights remained above 80%, suggesting that Na⁺ concentration had a minimal effect on the retention of TPPs. However, the ACE inhibitory activities of the peptides with three different molecular weights exhibited an initial increase followed by a decline, indicating a negative correlation between NaCl concentration and ACE inhibition rate, which aligns with the findings of Wang et al. [49] This phenomenon may be attributed to the high concentrations of NaCl reducing the solubility of proteins or peptides, resulting in flocculation or aggregation and consequently diminishing their activities [50].

### 3.4. Effect of pH on the Stability of Peptides with Varying Molecular Weights

Figure 5d,e show the effects of various pH treatments on TPP retention and ACE inhibition. The results indicate that peptides of different molecular weights retained >70% under all pH conditions, demonstrating good pH stability. In strongly acidic environments, all three peptides maintained >50% ACE inhibition, while in alkaline environments, inhibition approached or exceeded 60%. However, we observed reduced ACE inhibition for the 3 kDa and 1–3 kDa peptides in strongly acidic and alkaline conditions, possibly due to racemization and deamination, leading to structural changes and decreased activity [51]. Despite this, all three peptides with different molecular weights retained approximately 50% inhibitory activity across all pH conditions; however, neutral conditions are recommended for packaging and storage.

### 3.5. Effect of Temperature on the Stability of Peptides with Varying Molecular Weights

The thermal stability of TPPs is illustrated in Figure 5f,g. The retention of TPPs remained above 85% within the temperature range of 4 to 60 °C, while ACE inhibition exceeded 60%. However, the inhibition and retention of the three peptides with different molecular weights exhibited a marked decline when the temperature exceeded 60 °C. This reduction may be attributed to the degradation of the secondary structure of the peptide chain resulting from the elevated temperature [52]. Notably, the retention rate of peptides with a molecular weight of less than 1 kDa was 82.58% at 100 °C, which was significantly higher than that of other components. This is analogous to the thermal stability observed in protein-derived antihypertensive peptides from Arthrospira platensis. Since ACE inhibitory peptides are of low molecular weight, heating has minimal impact on their structure and stability [53]. Although low-molecular-weight peptides exhibit greater resistance to degradation at elevated temperatures, the detrimental effects of high temperatures on TPP biological activity necessitate avoiding such conditions during storage, processing, and preparation.

### 3.6. Effect of Organic Solvents on the Stability of Peptides with Varying Molecular Weights

**Figure 6:**
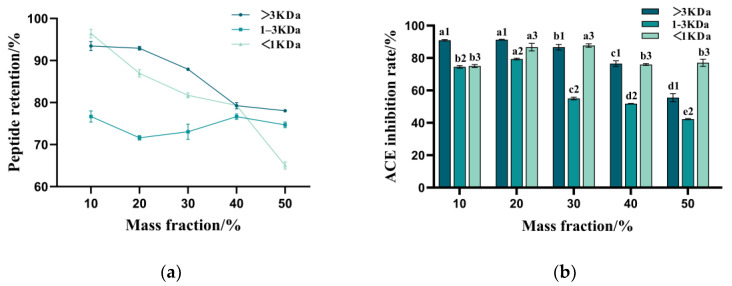

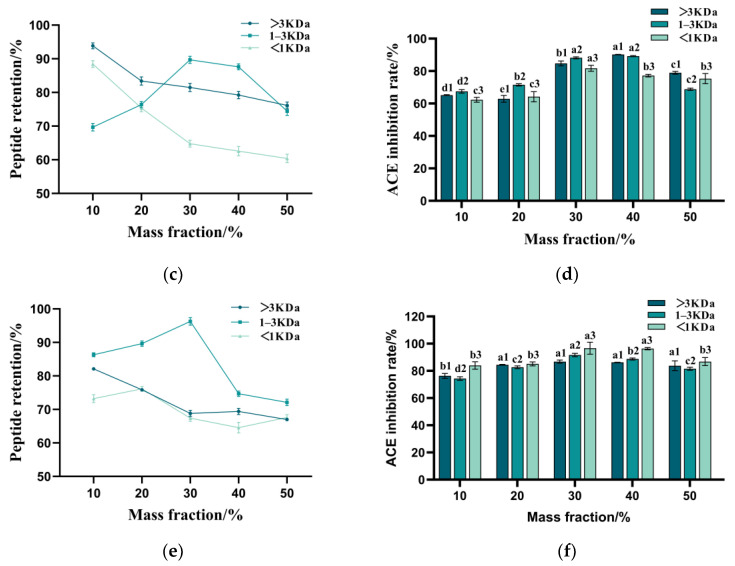
(**a**,**c**,**e**) Changes in ACE inhibition of samples after treatment with different mass fractions of methanol, ethanol, and propanol. (**b**,**d**,**f**) The variations in ACE inhibition in samples treated with different mass fractions of methanol, ethanol, and propanol are shown separately. Different letters indicate statistically significant differences among treatments for each sample (*p* < 0.05).

The retention of peptides from TPPs exhibited a gradual decline with increasing concentrations of methanol, ethanol, and glycerol (Figure 6). Notably, a slight increase in the concentration of TPPs with molecular weights ranging from 1 to 3 kDa was observed. This phenomenon may be attributed to the reduction in the dielectric constant of the aqueous solution upon the addition of organic solvents, which enhances the electrostatic attraction between dipole ions and decreases protein solubility. Additionally, it is plausible that the hydrophobic layer on the surface of protein molecules is compressed, leading to dehydration condensation among protein molecules. This process may impede protein decomposition and subsequently increase peptide content [54]. When the concentration of the organic solvent exceeds 30%, the ACE inhibition rate significantly declines. This reduction may be attributed to the dehydration and aggregation of proteins, resulting in the encapsulation of hydrophobic and aromatic amino acids, which possess functional activity, by hydrophilic amino acids. Consequently, this encapsulation leads to a decrease in ACE activity. Therefore, it is advisable to minimize exposure to organic solvents such as methanol, ethanol, and propanol, during the processing, storage, and transportation of TPPs.

### 3.7. Effect of Metal Ions on the Stability of Peptides with Varying Molecular Weights

**Figure 7:**
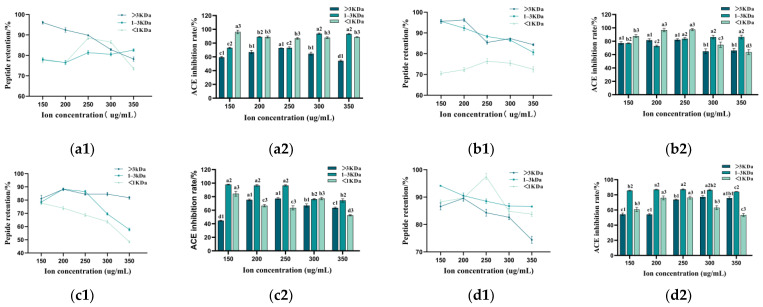
Effect of various metal ions on the stability of tomato pomace peptides with different molecular weights. (**a1**,**a2**) Changes in peptide retention and ACE inhibition in samples treated with different concentrations of K^+^. (**b1**,**b2**) Changes in peptide retention and ACE inhibition in samples treated with different concentrations of Mn^2+^. (**c1**,**c2**) Changes in peptide retention and ACE inhibition in samples treated with different concentrations of Fe^2+^. (**d1**,**d2**) Changes in peptide retention and ACE inhibition in samples treated with different concentrations of Cu^2+^. Different letters indicate statistically significant differences among treatments for each sample (*p* < 0.05).

Various metal ions significantly influenced the activity of tomato peptides during food processing, storage, and transportation (Figure 7). The retention of peptides and the ACE inhibition of tomato pomace decreased to varying extents following the addition of metal ions. This phenomenon may be attributed to the disruption of chemical bonds between peptides by the metal ions, leading to reduced solubility and increased exposure of hydrophobic groups. The effects of various metal ions on the ACE inhibition of peptides with differing molecular weights were ranked as follows: Fe^2+^ > Cu^2+^ > Mn^2+^ > K^+^. The addition of Fe^2+^ resulted in a decrease in ACE inhibition to 63.51%, 74.52%, and 52.5%. This reduction may be attributed to the disruption of chemical bonds between TPPs by the metal ions, which weakens the ionic interactions among peptides or leads to the chelation of metal ions with peptides, thereby affecting the biological activity of TPPs [54]. This indicates that metal ions exert a notable influence on the stability of TPPs, and it is advisable to minimize contact with metal material, especially those containing Fe^2+^ during the processing, storage, and transportation of TPPs.

### 3.8. Effect of In Vitro Simulated Digestion on the Stability of Peptides with Varying Molecular Weights

The gastrointestinal tract represents a significant barrier to bioactive peptides, with digestive enzymes playing a crucial role in influencing peptide bioactivity and bioavailability [55]. In vitro, simulated gastrointestinal tract digestion experiments were conducted to evaluate the stability of the prepared TPPs following gastrointestinal digestion (Table 4). The results indicated that during the gastric digestion phase, the ACE inhibition rate of peptides less than 1 kDa was 64.37 ± 7.24%, with a peptide retention rate of 84.88 ± 1.93%, significantly surpassing that of other components. However, a reduction in the ACE inhibition rate was observed compared to the blank group, likely attributable to the hydrolysis of tomato peptides into lower-molecular-weight peptides by pepsin. Additionally, more hydrophobic amino acid side chains are exposed, increasing the contact area between the digestion products and the enzyme, which enhances enzyme activity inhibition [56]. During the intestinal digestion phase, alterations in pH and degradation by trypsin compromised the key amino acid sequences associated with antihypertensive activity, leading to a 10.23% reduction in peptide retention. However, ACE inhibition remained relatively stable, potentially due to the presence of free amino acids. The peptide chains greater than 3 kDa exhibited less stability compared to the 1–3 kDa and less than 1 kDa fractions, likely due to their more complex structures. Enzymatic hydrolysis may further modify their amino acid sequences and structures, thereby diminishing ACE activity. Overall, the TPP demonstrated high stability during in vitro simulated gastrointestinal digestion, thereby providing a theoretical foundation for its application in functional diets [57].

### 3.9. Structural Characterization of Peptides

TPPs were structurally characterized using UV-Vis and FT-IR spectroscopy and scanning electron microscopy.

#### 3.9.1. UV

**Figure 8:**
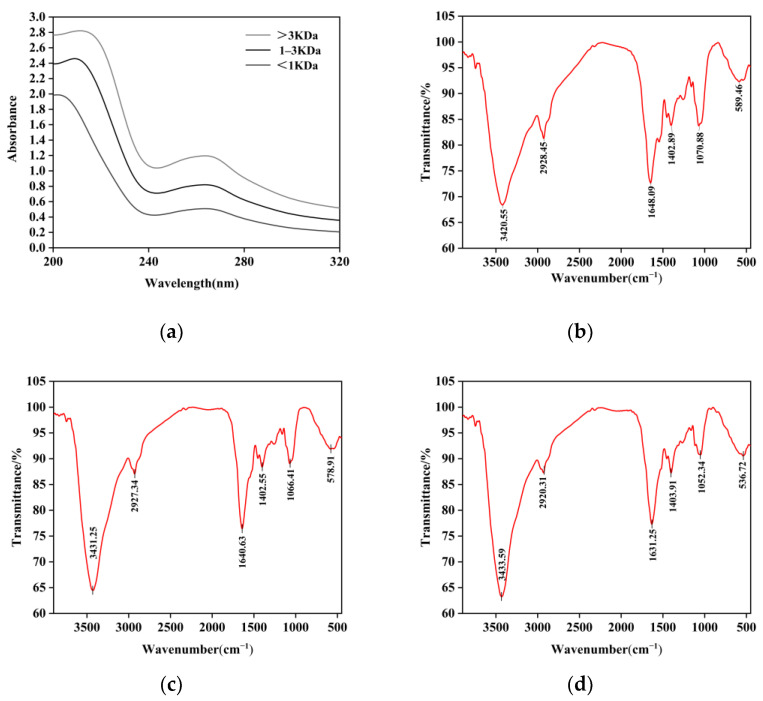
(**a**) UV spectrum of three molecular weight components. (**b**) Fourier infrared spectrum of components with molecular weights greater than 3 kDa. (**c**) Fourier infrared spectrum of components with molecular weights between 1 and 3 kDa. (**d**) Fourier infrared spectrum of components with molecular weights less than 1 kDa.

Figure 8a shows the UV absorption spectra of TPP fractions with different molecular weights. Peptides in the >3 kDa, 1–3 kDa, and <1 kDa ranges exhibited maximum absorption peaks at 212 nm, 209 nm, and 202 nm, respectively, primarily due to their tryptophan and tyrosine residues [58]. Additionally, the presence of peptide backbones and aromatic amino acids is further confirmed by the TPP’s absorption peaks at approximately 210 nm and 263 nm. This is in agreement with the results of Hong et al. [59] who studied tilapia peptides with a maximum absorption wavelength of 269.84 nm.

#### 3.9.2. FT-IR

FT-IR spectroscopy is a highly effective and sensitive method for assessing protein conformation [60]. The FT-IR spectra of TPP are presented in Figure 7b–d. The absorption peaks observed at 3420.55 cm⁻^1^, 3431.25 cm⁻^1^, and 3433.59 cm⁻^1^ are attributed to N-H stretching vibrations, while the characteristic peak near 2920 cm⁻^1^ is primarily associated with C-H vibrations. The absorption peaks at 1648.09 cm⁻^1^, 1640.63 cm⁻^1^, and 1631.25 cm⁻^1^ correspond to the amide I band. Previous reports [61] indicate that absorption peaks in the range from 1650 to 1660 cm⁻^1^ are associated with α-helical structures, whereas absorption peaks between 1600 and 1640 cm⁻^1^ correspond to β-sheet structures [62]. This suggests that TPPs predominantly exhibit a dense β-sheet conformation. The β-sheet conformation is a common secondary structure of proteins formed by a series of amino acid chains interconnected by hydrogen bonds, and the presence of a β-sheet is critical to its stability and function [63]. The absorption peak observed near 1400 cm⁻^1^ is attributed to the stretching vibration of C-N in amide III. These results further substantiate the presence of a peptide-specific structure in TPPs. In comparison to the fractions exceeding 3 kDa, the peak intensities of the other fractions were significantly higher; however, the peak positions exhibited minimal variation, indicating that the ultrafiltration separation and purification process did not compromise the overall structure of the tomato peptide.

#### 3.9.3. SEM

**Figure 9:**
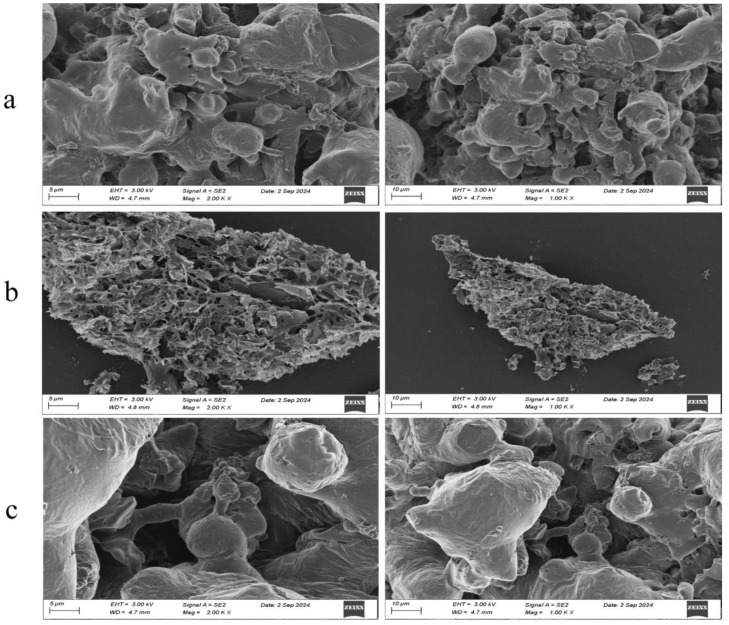
Scanning electron microscopy images of components with different molecular weights: (**a**) >3 kDa; (**b**) 1–3 kDa; (**c**) <1 kDa.

Scanning electron microscopy (SEM) was used to investigate the microstructural characteristics of tomato peptides with varying molecular weights (Figure 9). Peptides in the 1–3 kDa range showed significantly greater surface fragmentation than the <1 kDa and >3 kDa fractions. At the same magnification, the <1 kDa and >3 kDa peptides exhibited irregular, smooth, and dense surfaces with numerous textures and pores. These results are consistent with those reported by Li et al. [64] for soybean meal peptides from solid-state fermentation. The observed similarity may be due to the action of proteases produced during fermentation. These proteases hydrolyze tomato proteins, exposing hydrophobic groups on the peptide surfaces and thus increasing their hydrophobicity. The findings of this study contribute to a deeper understanding of the differences in the physicochemical properties of tomato peptides and offer valuable insights for future research on metal chelates involving tomato peptides.

## 4. Conclusions

This study introduces an effective method for the preparation of TP products with a high peptide content, achieved through a two-stage fermentation process. This method integrates yeast fermentation with lactic acid bacteria fermentation, resulting in a significant enhancement of peptide content while ensuring that the molecular weight of the peptides is predominantly concentrated within the small-molecule range. The stability of the peptide products obtained from the final isolation and purification process was evaluated. The presence of the obtained peptide was confirmed through UV and FT-IR analyses, which indicated that its secondary structure predominantly comprised a β-sheet configuration, further revealing its possible bioactive profile. This study provides a theoretical basis for the industrial production (especially processing and storage) of tomato peptides, which can be optimized in the future by response surface methodology and other optimized processes to improve yield and economic efficiency. In conclusion, tomato pomace peptides have the potential to be used in the development of functional foods (e.g., ACE inhibitors), but their mechanism of action needs to be further investigated, their bioactivities need to be verified by cellular and animal experiments, and more applications (e.g., beverages) need to be developed. Finally, to ensure the reliability of the results, it is recommended to repeat the experiments at different time points and environmental conditions to verify stability and reproducibility.

## Figures and Tables

**Table 1:** Determination of ACE inhibitory activity.

Additions	Sample Wells/μL	Blank Wells/μL
ACE (0.1 U/mL)	10	10
FAPGG (1 mmol/L)	50	50
ACE Inhibitors	40	0
HEPES	0	40

**Table 2:** Molecular weight distribution of peptides derived from the hydrolysis of tomato pomace by different bacterial taxa.

Sample	Molecular Weight (%)			
	<1 KDa	1–3 kDa	3–10 kDa	>10 kDa
*Bifidobacteria thermophilus*	91.08%	7.27%	1.46%	0.19%
*Lactobacillus rhamnosus*	89.43%	8.18%	2.13%	0.26%
*Pediococcus acidilactici*	88.89%	8.53%	2.3%	0.29%
*Bacillus subtilis*	92.15%	6.2%	1.44%	0.2%

**Table 3:** Amino acid composition of peptides fermented by four different strains.

Name/(mg/mL)	*B. thermophilum*	*L. rhamnosus*	*Bacillus subtilis*	*Pediococcus acidilactici*
Asp	1.3618 ± 0.0006	1.0033 ± 0.0028	1.2244 ± 0.0019	1.2053 ± 0.0019
Glu	2.3965 ± 0.0106	2.0211 ± 0.0010	2.3134 ± 0.0019	2.2077 ± 0.0070
Ser	0.5977 ± 0.0019	0.4128 ± 0.0090	0.5135 ± 0.0012	0.5191 ± 0.0005
His	0.2511 ± 0.0011	0.1691 ± 0.0004	0.2188 ± 0.0021	0.1991 ± 0.0007
Gly	0.8357 ± 0.0010	0.6106 ± 0.0012	0.7393 ± 0.0223	0.7913 ± 0.0047
Thr	0.4573 ± 0.0065	0.3357 ± 0.0041	0.3872 ± 0.0009	0.3896 ± 0.0008
Arg	0.7414 ± 0.0006	0.4917 ± 0.0011	0.6355 ± 0.0009	0.6251 ± 0.0012
Ala	0.6417 ± 0.0016	0.5118 ± 0.0102	0.5752 ± 0.0007	0.5908 ± 0.0010
Tyr	0.4072 ± 0.0062	0.2797 ± 0.0056	0.3375 ± 0.0007	0.3547 ± 0.0057
Cys	0.0789 ± 0.0089	0.0539 ± 0.0014	0.0703 ± 0.0011	0.0671 ± 0.0013
Val	0.4572 ± 0.0109	0.3298 ± 0.0060	0.4136 ± 0.0001	0.4167 ± 0.0009
Met	0.0842 ± 0.0010	0.0637 ± 0.0007	0.0814 ± 0.0024	0.0854 ± 0.0017
Phe	0.5579 ± 0.0023	0.3736 ± 0.0009	0.4563 ± 0.0007	0.4495 ± 0.0008
Ile	0.3873 ± 0.0080	0.2355 ± 0.0010	0.2960 ± 0.0096	0.2989 ± 0.0013
Leu	0.7075 ± 0.0009	0.5037 ± 0.0018	0.6481 ± 0.0012	0.6289 ± 0.0012
Lys	0.6923 ± 0.0055	0.4623 ± 0.0129	0.6041 ± 0.0013	0.5873 ± 0.0009
Pro	0.6948 ± 0.0028	0.5355 ± 0.0047	0.4688 ± 0.0011	0.5947 ± 0.0012
The sum of non-essential amino acids	7.2696 ± 0.0190	5.5868 ± 0.0299	6.4702 ± 0.0300	6.5341 ± 0.0176
Total essential amino acids	4.0809 ± 0.0512	2.8070 ± 0.0348	3.5134 ± 0.0200	3.4772 ± 0.0153
Hydrophobic amino acid content	3.5306 ± 0.0274	2.5536 ± 0.0254	2.9395 ± 0.0158	3.0649 ± 0.0082
Total amino acid content	11.3505 ± 0.0702	8.3938 ± 0.0646	9.9836 ± 0.0500	10.0113 ± 0.0329

**Table 4:** Effect of pepsin and trypsin on peptide retention and ACE inhibition. Different lowercase letters indicate significant differences (*p* < 0.05).

	ACE Inhibition Rate (%)		Peptide Retention (%)	
	Gastric Digestion	Intestinal Digestion	Gastric Digestion	Intestinal Digestion
Blank	72.65 ± 0.28 a	72.87 ± 5.56 a	-	-
<1 kDa	64.37 ± 7.24 ab	66.42 ± 3.85 ab	84.88 ± 1.93 a	73.74 ± 2.78 a
1–3 kDa	62.66 ± 2.09 ab	61.33 ± 1.98 ab	81.91 ± 1.6 ab	72.73 ± 2.09 ab
>3 kDa	59.24 ± 3.57 b	57.81 ± 9.08 b	79.12 ± 0.69 b	68.48 ± 0.97 b

## Data Availability

The original contributions presented in this study are included in the article. Further inquiries can be directed to the corresponding author.
